# Needs and early response towards internally displaced people in Hatay Province in the aftermath of the 2023 earthquake in Turkey

**DOI:** 10.1186/s12889-025-22501-0

**Published:** 2025-05-08

**Authors:** Guenter Froeschl, Buse Zagli, Ege Erucar, Kutaiba Alaaeddin, Mustafa El Othman, Cem Hatunoglu

**Affiliations:** 1 Institute of Infectious Diseases and Tropical Medicine University Hospital, LMU Munich, Munich, Germany; 2 Dünya Doktorlari Dernegi, Doctors of the World Turkey, Istanbul, Turkey; 3 Division of Infectious Diseases and Tropical Medicine, University Hospital, LMU Munich, Munich, Germany; 4 Altınbaş University Faculty of Medicine Bahçelievler Medicalpark Hospital, Istanbul, Turkey

**Keywords:** Turkey, Hatay, Earthquake, 2023, Humanitarian, Response

## Abstract

**Background:**

On 06 February 2023, an extreme earthquake affected Hatay province in Turkey. The immediate response consisted of medical teams focusing on traumatology and immediate trauma related care. In the intermediate time period, after days to weeks, more primary health care was needed for displaced populations in tent shelters and in surrounding villages. We are describing the results of a needs assessment intervention and health services provided by an international non-governmental organization.

**Methods:**

Mobile teams circulated to displaced populations in peripheral locations. Fourty-two representatives of communities were interviewed for rapid needs assessment. After triaging and identification of needs, mobile medical units offered primary health care services. Data was collected digitally, directly by the healthcare workers on basic demographics and health conditions. We are reporting a descriptive overview of the data.

**Results:**

Communities showed different degrees and dimensions of need, such as entirely lacking health services, or missing sanitary facilities. Populations in the communities increased in most sites. From 16 February to 06 April 2023, 3,027 patients were attended to. The majority of our beneficiaries were female (61.0%) and of Turkish origin (66.9%). Children under the age of 18 accounted for 41.3%. The most reported health findings were upper respiratory infections (24.9%) and scabies (9.7%). In 68 patients, a primary diagnosis of a mental health condition was made.

**Conclusions:**

In the intermediate response after an earthquake-driven disaster, primary healthcare provision becomes a crucial element of humanitarian support. Massive displacement into crowded tent shelters lead to respiratory conditions and contagious ectoparasitism. Organizations engaging in this context need to be prepared accordingly, not the least by sufficient stock keeping.

## Introduction

On 06 February 2023, at 04:17, an earthquake with a magnitude of 7.7 occurred in the Pazarcık district of Kahramanmaraş, Türkiye, followed nine hours later by a second shock of almost the same magnitude [1]. In the days following these first two prominent shocks, nearly 17,000 aftershocks were registered, affecting also the humanitarian workers that came to the region to support the population. Affected by this massive earthquake were also the bordering regions of Syria. According to reports by UN-OCHA and Reliefweb, in Turkey more than 200,000 buildings collapsed, more than 50,000 people lost their lives, more than 100,000 were injured, and 3 million were displaced, while in Syria more than 2,000 buildings were destroyed, 5,900 people were killed and 350,000 were displaced [2, 3]. In Türkiye, the disaster affected the provinces Kahramanmaraş, Hatay, Gaziantep, Malatya, Diyarbakır, Kilis, Şanlıurfa, Adıyaman, Osmaniye and Adana, and a state of emergency was declared by the national government in these provinces [3].

In a census by the Turkish Statistical Institute, the population of Turkey in 2022 was indicated as 85 million people. According to the same census data, the population of the 10 most affected provinces is 13 million people, which translates into one out of six of Turkish citizens directly being affected by the earthquake. In the affected provinces, already prior to the earthquake, there was also an estimated 2 million refugees, mostly from Syria [3, 4].

The immediate response consisted of finding and evacuating survivors from rubble, and providing basic support for the survivors. Therefore, a focus was placed on search and rescue teams, including the use of sophisticated equipment, and critical care for evacuated survivors. In addition, survivors had to be catered with basic shelter, food and water [5]. The sheer number of affected survivors, with an estimated 3 million displaced people, resulted in a challenge regarding the volume of basic service items [3]. In this early phase, many informal shelters were set up, at roadsides or on any other kind of vacant space, even though the delivery of tents was scaled up quickly. Also, health services were mostly set up in tent structures, that were geared towards treating injuries, hence comprising facilities in surgery, anaesthesiology and intensive care, such as the field hospital on the premises of the heavily damaged state hospital in Antakya. The coordination of the early phase was covered by Turkish state authorities, and were executed under involvement of the Turkish governmental disaster relief organization AFAD (Afet ve Acil Durum Yönetimi Başkanlığı) [5, 6]. However, also numerous international actors were involved, such as missions from larger humanitarian organizations or delegations from other countries. Examples are emergency facilities deployed by the governments of Belgium, Indonesia and Spain [7, 8].

The early phase shifted over to the intermediate phase, when the probability of finding survivors became minimal, roughly between week one and two after the earthquake. In order to provide improved shelter, and to facilitate more centralized services in terms of health care, food and water provision, and of sewage management, larger clearings were prepared, where so-called tent- or container-cities were set up, with uniform rows of shelters, with capacities to house several hundreds of individuals. These shelters were mostly found at the outskirts of the urban areas. Here, also telecommunication services and activities for children, such as playgrounds or mobile libraries, were prepared. In these structured facilities it was mostly the government that took responsibility, also for health care services. As for mental health, mostly services addressing post traumatic stress disorder were introduced, for example by the Turkish Red Crescent society [9].

Support for the displaced population that had fled out of the urban area and into the rural areas in the surrounding mountainous region was even more challenging. Some of the displaced individuals from urban areas are actually originating from surrounding villages or have relatives there. Many of the mostly agricultural villages increased sharply in population size. Both autochthonous population and the displaced population remained sheltered in rather informal tents, scattered across villages. Locally available infrastructure, if still intact, was overcharged by the increased population size. The scattered population and the regions that were difficult to reach, became a challenge to relief operations.

Dünya Doktorlari Dernegi (DDD) - the Turkish section of Médecins du Monde/ Doctors of the World, was established in Turkey in 2015 [10]. Prior to the earthquake, it had implemented programmes in both Turkey and Syria, offering free access to healthcare services to refugees and internally displaced populations due to the conflict in Syria. These services comprised, next to basic primary healthcare services, also emergency outpatient care, mother- and child-care, and psychosocial support through psychologists and protection officers trained to attend to vulnerable people [11]. The organization had therefore already an established base in Antakya and can be considered a local player. DDD was therefore present in the urban centre of Antakya when the earthquake took place, and was suffering also substantial losses, such as own staff being killed or injured, and its office building being entirely destroyed [12]. The organization has additional headquarters in Istanbul and Izmir, and after a brief period of re-organization, it was able to take up its work again, with a container-based structure that was set up in the centre of Antakya, and with mobile teams in vehicles.

Since DDD is familiar with the region, and disposes of teams that are trained to work under mobile conditions, the organization took the delivery of health care services to the scattered villages in the mountainous regions as its mandate. This study is presenting the findings from an analysis of the operational data collection of the mobile medical units. The aim of this study is to describe the healthcare needs of a population after a massive displacement by an extreme earthquake. The data covers the intermediate response time period, from ten days to nine weeks after the incident.

## Methods

### Study design

This is a descriptive, cross-sectional and retrospective study. We used data from a digital surveillance database. the open source platform DHIS2 (HISP Centre at the University of Oslo, Norway) [13].

### Setting

The study was conducted in Hatay province in the South-West of Turkey, one of the most affected areas of the earthquake of 06 February 2023. The analysis is based on the data collected as part of a healthcare intervention by an international non-governmental organisation, Dünya Doktorlari Dernegi, which is the chapter in Turkey of Médecins du Monde. The earthquake response started immediately after the event, however, systematic data collection only started after ten days, when primary healthcare needs of displaced populations was moving into focus. The data used in this analysis comprises the time period from 16 February (beginning of data collection) to 06 April 2023.

The humanitarian organisation is located and operational in the region since 2016, serving the needs of Syrian refugees to the region. It was operating its post-earthquake activities from a container-based emergency base in the centre of Antakya, the capital of Hatay province, since its previous operational centre was completely destroyed in the earthquake. From there, the organisation was setting up Mobile Medical Units that were travelling daily to displaced populations in areas away from Antakya, as the city itself was practically uninhabited after the massive displacements. The locations visited are mostly rural and situated in mountainous regions, some 30 to 120 minutes by car.

The Mobile Medical Units consisted of a medical doctor, one nurse or midwife, one psychologist, one case-worker, one so-called protection officer, one translator and a driver which also served as crowd-controller. The teams carried medicines in pre-prepared containers. The units’ deployments occurred in minivans, allowing movement also on rough and narrow roads. During the reported period, two Mobile Medical Units were operational. The equipment of each unit comprised a mobile pharmacy with medicines for the most common communicable and non-communicable diseases, tools for taking vital parameters; the data collection was executed digitally via the DHIS2 application installed on mobile phones of the unit leader.

The deployments served primarily two activities that were partly occurring simultaneously, partly in separate deployments. The activities were Site Assessments and Healthcare Surveillance Data collection.

### Study population

The study population consisted of both the displaced population and of the hosting communities. The background population size is difficult to state due to the massive displacement that had taken place. However, according to the Turkish statistical office the population of Hatay province was almost 1.7 million, the population of Antakya city almost 400,000 in 2022 [4]. The geographical locations that were included in the study were identified by the study team through exploratory field trips along accessible roads in the mountain areas surrounding Antakya city. In some cases the study team received mobile phone messages from community representatives requesting support. As much as possible, the assessment teams tried to interview official representatives (mukhtars: mayor), but in cases where these were absent also other persons that were accepted by the community as spokespersons were interviewed.

For the Healthcare Surveillance Data analysis, the population in an identified settlement of need was informed by the representative the day before the intervention, mostly by announcements via loudspeakers from mosques or mayors’ houses. The patients were then seen consecutively by medical doctors or nurses of the study team, upon their appearance at the intervention site of the Mobile Medical Unit. Line-up was secured by crowd-controllers, mostly by issuing numbers, or by collecting identity cards and reading out names.

### Tools

Data was entered directly by staff into an online database, using the open source platform DHIS2 (HISP Centre at the University of Oslo, Norway) [13]. Two questionnaires were used, the Site Assessment, and the Healthcare Surveillance Data questionnaire. The questionnaires are added as Supplementary Files.

Data of the Site Assessments was entered through a data entry form, covering GPS location, classification of the site as an urban or a rural setting, information on the contact person, pre- and post-earthquake population numbers, estimation of severely damaged and uninhabitable buildings, existing health care provision, water, food and sanitation situation. As urban or semi-urban were defined all settings in the urban plains of Antakya, Defne, Kirikhan and Samandag, which represent larger metropolitan areas. Rural villages were separated settlements, detached from the metropolitan areas, and with populations of fewer than 10,000. These were furthermore classified by district, which are Altinözü, Antakya, Defne, Kirikhan, Kumlu and Samandag.

The Healthcare Surveillance Data of patients was entered directly during the medical visits through a separate entry form into a separate database, using the same platform DHIS2. The health surveillance data comprised age, sex, origin, chronic conditions, symptom and diagnosis checklists, and the services provided. The questionnaires were self-designed by the team, supported by a researcher, experienced in humanitarian aid and data analysis. The variables on “medicine delivered”, “dressing applied”, “internal referral” and “external referral” were added and operational on 08 March 2023, hence data on these items are given only for the time period thereafter. Age was generally collected as per age groups: 0- <5 years; 5 to <18 years; 18 to <65 years; >=65 years. On 17 March 2023, the variables age in months and age in years were added. Age in months was then transformed into decimals of age in years.

### Statistical analysis

Data was exported from the DHIS2 databases into Excel spreadsheets (Microsoft Corporation, Redmont, USA), and these imported into Stata 15SE (Stata Corporation, College Station, USA). Descriptive categorical data is presented in frequencies and proportions, continuous data in median and quartiles. Analysis of correlation were executed by application of Chi^2^ Tests for categorical data, and by using Student t-Test for continuous data.

## Results

### Site assessments

**Fig. 1:**
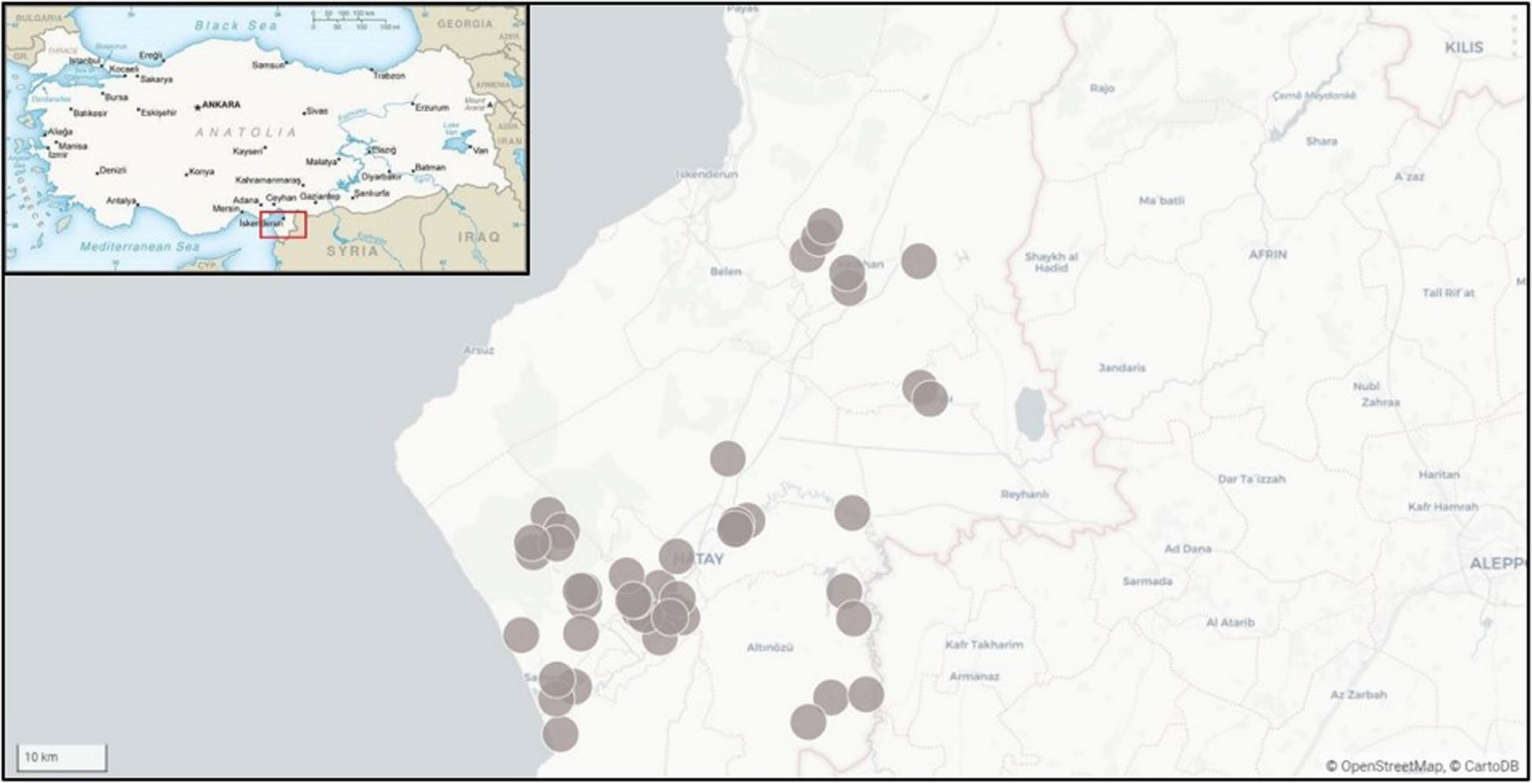
Map of Hatay Province and Visited Sites, Hatay Province, 16 February to 06 April 2023

The site assessment teams investigated 42 locations in 6 districts of Hatay province. Figure 1 is indicating the geographical layout of Hatay province and the districts that were served by the Non-Governmental Organization. In the map, the individually assessed locations are indicated by grey circles.

The grey circles refer to all sites that were assessed by the assessment teams. In the inserted top left corner is a map of Turkey with a red box indicating the location of the larger image within Turkey (large map is an output from DHIS2; inserted map is based on the Turkey base map with permission to use from the US CIA) [14].

The interviewees were in 47.6% (20/42) of cases the mayors of the locations (mukhtar), in the other cases other representatives or informed individuals, such as teachers. The needs assessments revealed that 67.6% (25/37) of the locations were suffering from substantial destruction of buildings themselves, defined as equal or more than 25% destruction of its buildings resulting in an uninhabitable condition. In general, urban settings were more damaged (mean 45% of buildings damaged) as compared to rural settings (mean 40%), although this difference was not significant. At the same time two sites showed signs of massive overpopulation with increases of more than 100% as compared to the pre-earthquake population (Table 1).

**Table 1:** Site Assessments, Population, Hatay Province, 16 February to 06 April 2023

District	Setting Type	Pre-Earthquake District Population*	Number of assessed locations	Pre-Earthquake Site Population	Post-Earthquake Site Population
Altinözü	Urban	60,000	0	n/a	n/a
Altinözü	Rural	60,000	6	1,000 to 1,300	1,600 to 6,000
Antakya	Urban	399,000	5	No data	30 to 1,000
Antakya	Rural	399,000	1	No data	1,600
Defne	Urban	165,000	4	900 to 3,000	600 to 3,200
Defne	Rural	165,000	7	800 to 1,100	750 to 8,000
Kirikhan	Urban	121,000	2	No data	110 to 450
Kirikhan	Rural	121,000	4	No data	200 to 1,700
Kumlu	Urban	13,000	1	950	950
Kumlu	Rural	13,000	0	n/a	n/a
Samandag	Urban	123,000	3	1,600	250 to 1,600
Samandag	Rural	123,000	9	200 to 15,000	15 to 16,000
Total		881,000	42		

Site populations refer to defined settlements

^*^district population is based on Turkish Population Statistics, rounded to thousands [15]. n/a: not applicable

Regarding the shelter, food, water and sanitation conditions, there were differences in trends between urban and rural locations. Occasional scattered tents were found more often in rural (16/27) as compared to urban (4/15) settings, whereas dwellings in camps was found more often in urban areas. Provision of food was more frequently done by the local autochthonous population in rural areas (12/27) as compared to urban settings (4/15). Surface water as type of water supply was only found in rural settings (7/27). Pre-existing sanitation facilities were more frequently still used in rural areas (18/27) as compared to urban areas (3/15), due to massive destruction in the latter.

The sites reported about the type of healthcare services available. In 19.5% (8/41) of the sites a local provider was available as before the earthquake. In 17.1% of sites (7/41) an external healthcare provider came already on a regular basis. In 43.9% (18/41) of sites, some external healthcare provider has already presented after the earthquake, but came only once or irregularly. In 19.5% (8/41) no service whatsoever was available. A part of the sites were classified by the assessors with “some need” for additional health services (36.6%; 15/41). In 43.9% (18/41) there was no need for additional services, which included all sites where there was a local doctor or already a regular visit by an external provider established. In 19.5% (8/41) there was a “high need”, mostly in the sites where no services were available.

### Healthcare consultations

The mobile medical units visited 36 locations, executing 3,027 healthcare consultations. Table 2 is giving an overview of the composition of the total beneficiary population.

**Table 2:** Socio-Demographic Characteristics of Patients by Origin, Hatay Province, 16 February to 06 April 2023

Origin	Number and proportion of total population	Age median (interquartile range), in years	Proportion Children < 5 years of age	Proportion Children 5 to < 18 years of age	Proportion Adults >= 65 years of age	Sex: proportion of females
Syrian	987/3,027 (32.6%)	21 (5 – 40)	215/987 (21.8%)	264/987 (26.8%)	77/987 (7.8%)	624/987 (63.2%)
Syrian with acquired turkish citizenship	14/3,027 (0.5%)	11 (4 – 12)	3/14 (21.4%)	5/14 (35.7%)	1/14 (7.1%)	8/14 (57.1%)
Turkish	2,026/3,027 (66.9%)	33 (10 – 55)	270/2,022 (13.4%)	492/2,022 (24.3%)	313/2,022 (15.5%)	1,213/2,026 (59.9%)
Total Population	3,027 (100%)	30 (9 – 53)	488/3,023 (16.1%)	761/3,023 (25.2%)	391/3,023 (12.9%)	1,845/3,027 (61.0%)

Proportions are given as row proportions within origin groups. Denominators may vary due to missing data for age

**Fig. 2:**
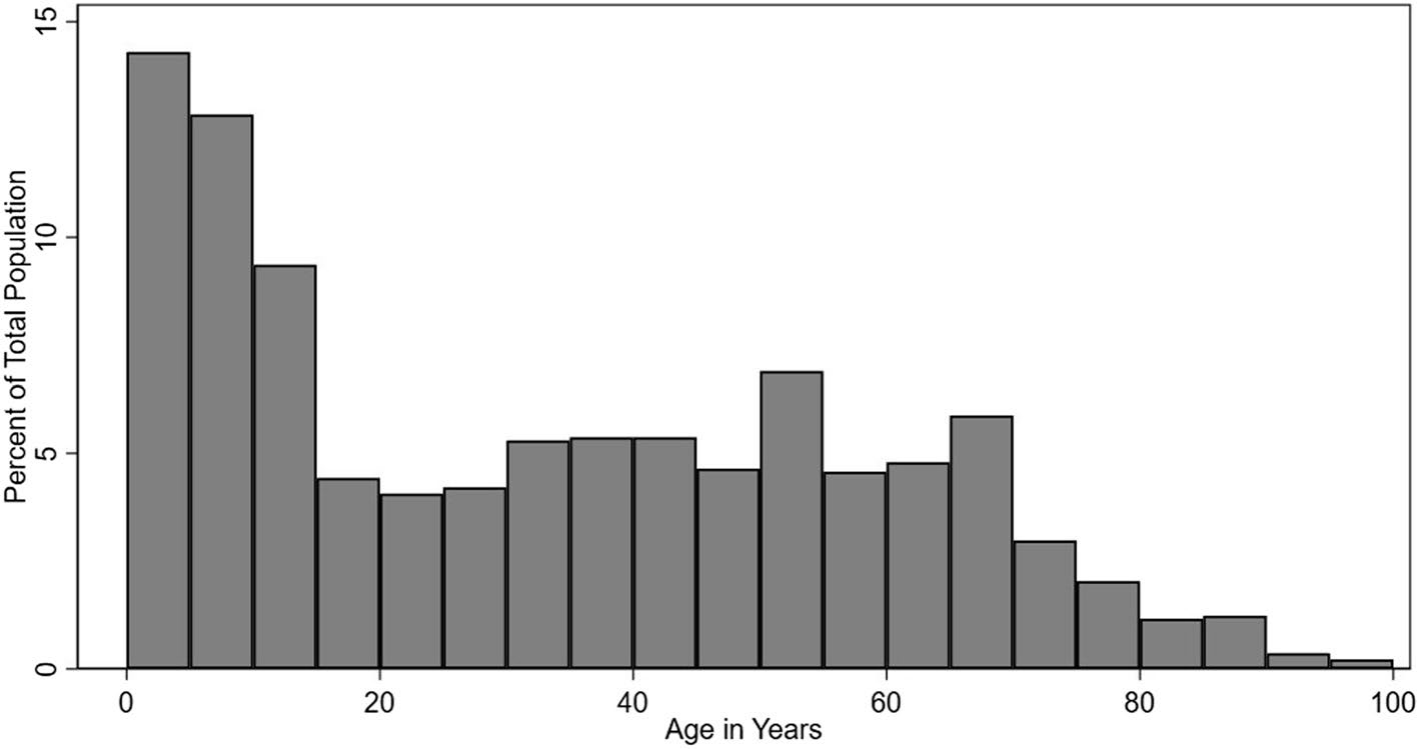
Age Distribution, Hatay Province, 16 February to 06 April 2023

The majority of patients visited were female with 61.0%, and of Turkish origin with 66.9% of all patients. About one third of the patients indicated to be of Syrian origin (33.1%), of which 1.4% (14/1,001) had already acquired Turkish citizenship. Children below the age of 5 years made up 16.1% of all patients, their share was higher in the subgroup of Syrian origin with 21.8% (*p* < 0.05). Furthermore, 12.9% of the population was aged 65 and above (Table 3 and Figure 2). A significantly higher share of patients aged 65 and above were found in the Turkish subgroup (*p* < 0.05).

**Table 3:** Reported Health Conditions, Total Population, Hatay Province, 16 February to 06 April 2023

Condition	Proportion	Cumulative Proportion
Upper Respiratory Infections	753/3,021 (24.9%)	24.9%
Scabies	292/3,021 (9.7%)	34.6%
Diarrhoea	227/3,021 (7.5%)	42.1%
Myalgia	183/3,021 (6.1%)	48.2%
Hypertension	148/3,021 (4.9%)	53.1%

The table shows the five most frequent conditions, with proportions and cumulative proportions referring to the total population

Histogram for proportion of age in years as per total population, bin width 5 years.

Most patients were reporting one health condition for which they sought services (2,684/3,027; 88.7%). However, in 343/3,027 (11.3%) there were multiple conditions reported. The share of patients with multiple diagnoses was significantly higher in the age group >= 65 years with 21.7% (85/391; *p* < 0.05). Table 3 gives an overview of the most commonly reported conditions as primary conditions for consultations.

The reported diagnoses varied by age groups. In children under the age of 5 years, the mostly reported conditions were upper respiratory infections and diarrhoea. Very prominent here was the prevalence of scabies, with 5.2% of all children in this age group showing clinical signs of scabies (Table 4).

**Table 4:** Reported Health Conditions, Children under Age of 5, Hatay Province, 16 February to 06 April 2023

Condition	Proportion	Cumulative Proportion
Upper Respiratory Infections	246/485 (50.7%)	50.7%
Diarrhoea	62/485 (12.8%)	63.5%
Dermatitis (allergic)	31/485 (6.4%)	69.9%
Scabies	25/485 (5.2%)	75.1%
Conjunctivitis	14/485 (2.9%)	77.9%

The table shows the five most frequent conditions, with proportions and cumulative proportions referring to the total population within the age group

In children under the age of 18 years, the leading reason for consultations remained upper respiratory infections, however, the second and third most frequent reason were scabies and lice as ectoparasites (Table 5).

**Table 5:** Reported Health Conditions, Children Age 5 to <18, Hatay Province, 16 February to 06 April 2023

Condition	Proportion	Cumulative Proportion
Upper Respiratory Infection	289/761 (38.0%)	38.0%
Scabies	123/761 (16.2%)	54.1%
Lice	53/761 (7.0%)	61.1%
Diarrhoea	51/761 (6.7%)	67.8%
Dermatitis (allergic)	40/761 (5.3%)	73.1%

The table shows the five most frequent conditions, with proportions and cumulative proportions referring to the total population within the age group

The most reported conditions in the elderly population of age 65 and above are of chronic and non-communicable nature. The most important diagnoses were hypertension and diabetes, which together made up 31.8% of all primary reasons for consultation. Next came myalgia with 11.5%. The fourth and fifth most frequent reasons were then again upper respiratory tract infections and diarrhoea (Table 6).

**Table 6:** Reported Health Conditions, Adults Age 65 and above, Hatay Province, 16 February to 06 April 2023

Condition	Proportion	Cumulative Proportion
Hypertension	74/390 (19.0%)	19.0%
Diabetes	50/390 (12.8%)	31.8%
Myalgia (mono-symptomatic)	45/390 (11.5%)	43.3%
Upper Respiratory Infection	27/390 (6.9%)	50.3%
Diarrhoea	23/390 (5.9%)	56.2%

The table shows the five most frequent conditions, with proportions and cumulative proportions referring to the total population within the age group

**Fig. 3:**
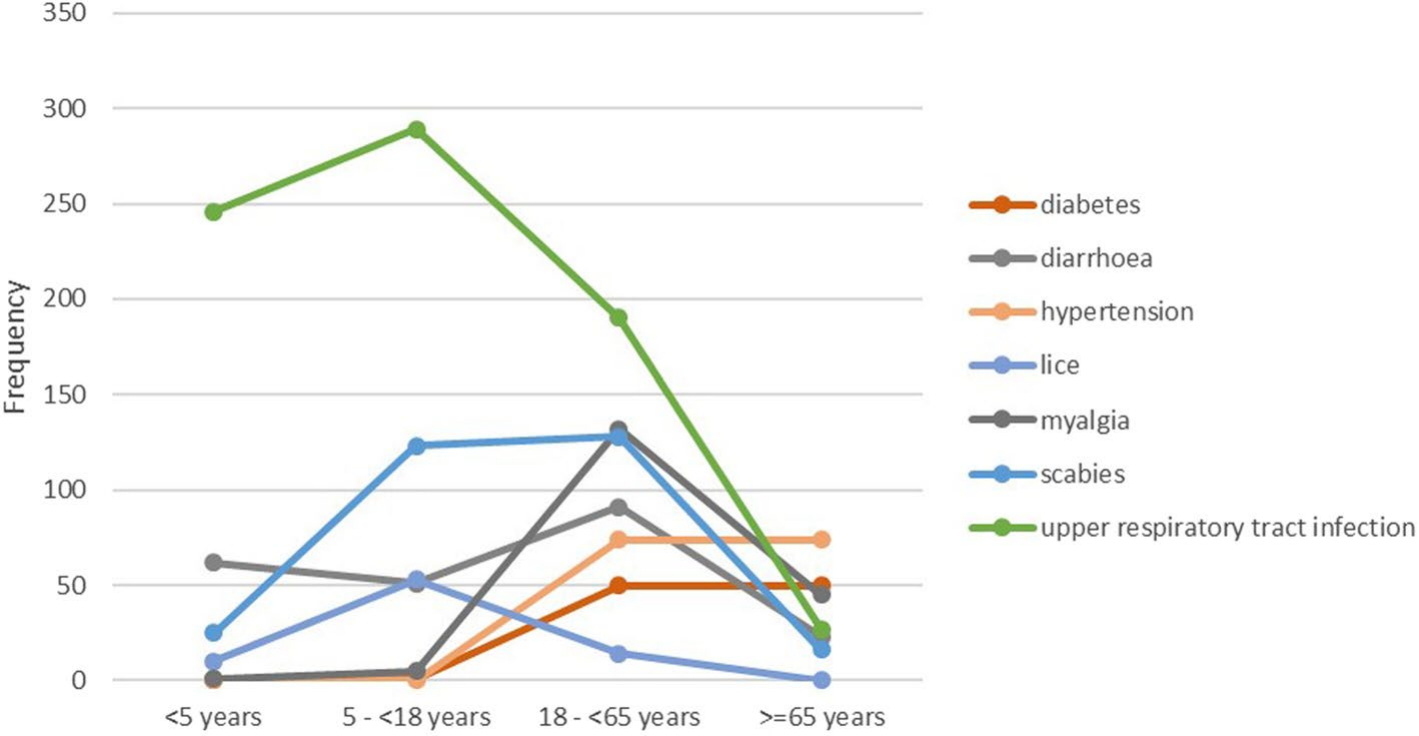
Frequency of Conditions across Age Groups, Hatay Province, 16 February to 06 April 2023

The variation in reported conditions across age groups is shown in figure 3. Here, the prominence of upper respiratory infections in the paediatric population can be appreciated, as well as the increase of chronic diseases (hypertension and diabetes) in older ages.

The lines are indicating the most frequently reported conditions across age groups. The y-axis is representing frequencies.

The largest proportion of healthcare needs could be served immediately by the attending healthcare worker. In 89.1% of patients, medicines were directly delivered for treatment. In 1.5% (33/2,257) of cases a referral was reported as necessary. For 33 patients (1.5%) surgical or wound dressings were applied.

Pregnancy was noted in 2.4% (22/921) of all females in the age group 18 to <65 years of age. Underage pregnancy was noted in 1.3% (6/451) of the female patients in the age group 5 to <18 years of age.

A mental condition was explicitly noted in 0.7% (21/3,021) of patients, among which in particular were noted depression in 10 cases and suspicion of post-traumatic stress disorder or anxiety in 11 cases. An internal referral towards the accompanying psychosocial support and protection officers was done in 20 cases.

Dental conditions, mostly caries, were reasons for consultations in 3.0% (91/3,021) patients, however, in the age group 5 to <18 years, 3.7% (28/761) were suffering from dental conditions.

## Discussion

The main findings of our study were a substantial unmet healthcare need in most of the visited populations. About one third of the population had a migration background with mostly Syrian origin. A majority was female, and more than 16% were children under the age of five. The most frequent diagnoses were unevenly distributed across the age groups, with upper respiratory tract infections and diarrhoeal diseases most prominent in children, and chronic non-communicable diseases in the elderly.

The earthquake of 06 February 2023 in Turkey is considered a severe earthquake-driven disaster. More than 50,000 individuals lost their lives, more than 100,000 individuals remained injured, and 3 million individuals were forced to displacement [2, 3]. Only in Hatay province, more than 21,000 are estimated to have died [16]. The displacements are at large internal displacements, mostly to surrounding areas. The immediate response was geared towards immediate lifesaving of survivors from the rubble, and relocation of survivors into temporary shelters, as has been reported also in other comparable disasters, such as the 2010 earthquake in Haiti [5, 17]. After few days to weeks, though, primary healthcare needs moved into focus, whereby a large displaced population has to be served [6]. The population was sheltered mostly in tent structures, and remained exposed to crowding, low temperatures and humidity. We saw most hosting communities facing a massive influx of displaced persons, overwhelming local infrastructure and services. We found numerous communities left dependent on external provision of drinking water and food items, and forced to resort to substandard hygiene practices, such as open defecation, and to risky water provision from surface water bodies. These immediate impacts on both displaced people and the hosting populations in internal displacements in disaster situations has also been described in other similar contexts [17, 18].

In addition, previously running healthcare services, with healthcare staff mostly living in the urban areas and higher-level services also being located in the same urban areas, came to an almost complete halt after the massive destruction of the urban areas. Therefore, our patient clientele comprised the entire range of the local population, comprising all age groups, but also pregnant women, and other people with special health needs, such as mostly elderly, chronically ill persons. In some situations, the deployed staff had to conduct home visits to attend to bedridden patients. The latter can put the service capacity of a mobile team at its limits, as it is heavily consuming time and staff as scarce resources. In some of the assessed sites, local healthcare providers were able to continue their service delivery as before the earthquake. However, they reported to us shortages of materials and medication (personal communications). Overall, we could also show that in the earthquake response some communities that were located in the periphery and the mountainous regions, were left underserved as compared to the larger tent or container sites adjacent to the affected cities. Humanitarian response should be monitored for such gaps, and activities be adapted accordingly. In cases where larger entities such as governmental organizations do not dispose of the capacity to de-centralize their efforts, independent humanitarian non-governmental organizations can fill these gaps [19].

In the numerous health visits, the attending healthcare staff had to engage also in health information and education (data not shown). This is particularly challenging but at the same time crucial in this emergency situation, even more than in regular primary healthcare situations, as many habits and behaviours need to be adapted to the substandard living conditions of the affected population in this humanitarian emergency situation [20]. For example, we saw many children with dental cavities, tooth brushing was either infrequent or completely abandoned in the aftermath of the earthquake, in most cases neither toothbrushes nor toothpaste were available. Another peculiarity of health literacy was the fixation of patients on brands of medicines. The notion of generic active agents is largely inexistent in the affected area. Hence, patients were in doubt and had to be informed about the importance and interchangeability of active agents and drug classes.

The observed morbidity profile revealed a prominence of upper respiratory tract infections that can be explained by the cold and humid environment at that time of the year, with temperatures approaching freezing point. Another factor is the crowded living conditions, with many being forced to practically sleep on the ground in the tents. Towards the end of the observation period, with increasing ambient temperatures, we could observe more diarrhoeal diseases. Also associated with crowded living conditions, we were confronted with ectoparasites such as lice and scabies, mostly in children, as has been reported elsewhere in comparable contexts [21]. At the same time, we as well as all healthcare providers active in the region, were faced with shortages of medications and other materials; we were repeatedly running out of stock, or had to execute class-changes in treatments of patients with chronic illnesses [22]. However, when available, the application of shampoos and creams was also a challenge in the crowded conditions with lacking privacy. In such large, displaced populations in a situation of an outbreak of scabies, we suggested to the local health administration the use of oral treatment for scabies with ivermectine, which was, however, not registered for this indication and not available at the time.

The Mobile Medical Units were able to serve a large number of the affected population, but had to do so under some limiting conditions. Possibilities for referral were very limited, e.g. to specialists in the field of cardiology, or to dentists. Specialist services were running also in temporary tent structures, but few in numbers, with low intake capacity. In addition, they were often difficult to access for the population in the mountain regions, as the services were again located in the lowland areas. In cases of suggested referrals, patients and their families were self-responsible to engage in the transportation or in calling a rudimentary ambulance service. In some few cases the MMU staff took over the calling of the ambulance service. A follow-up of patients for whom we have recommended referral was not possible, we are therefore unable to assess the efficacy and quality of referral treatment.

Our study is subject to some limitations. The scope, detail and breadth of the collected data is limited, as the data entry had to be conducted in situations of high patient turnover by the healthcare providers. Diagnoses were based mostly on clinical judgement, and in a smaller part of patients on clinical examinations, using senses, a stethoscope and an otoscope. Laboratory investigations were not available at all. Proper stock-keeping procedures for the drugs, that came in irregularly through private and informal donations, was not possible during the reported period. As the online data collection tool was improved and adapted over time, some of the variables were only added at a later stage. This was laid out in the methods section.

Furthermore, we are unable to highlight how equitable the access to the services were. A part of the population did not speak the Turkish language. However, also the local Turkish population in this region is frequently using Arabic as their mother tongue, and local passing of information is taking place both in Turkish and Arabic likewise [23]. In how far the localised community mobilisation may have led to the systematic exclusion of some parts of the population, we are unable to say.

The collection of surveillance data is essential, also and especially in humanitarian contexts of disasters [20]. We were able to install and operationalize an online data collection tool that allowed us to compile data that is crucial for the characterization of our target populations, and for the operationalization of the mobile units and for stock keeping of drugs. Furthermore, we would like to point out the massive needs of both hosting and displaced populations in terms of basic primary healthcare needs, as these populations are at large left without any corresponding healthcare services in the aftermath of a disaster of this extent. Organizations engaging in humanitarian support in such a phase need to be prepared for a high patient turnover, and need to be equipped with staff that is sufficiently resilient and trained for attending patients under circumstances of limited resources.

## Data Availability

The data that support the findings of this study are available from the corresponding author, but restrictions apply to the availability of these data, which were collected by the humanitarian organization Dünya Doktorlari Dernegi (Méecins du Monde Turkey) and analysed retrospectively for the current study, and hence are not publicly available. Data are however available from the corresponding author upon reasonable request and with permission of Dünya Doktorlari Dernegi.
